# Evaluation of nutritional profile and total antioxidant capacity of the Mediterranean diet of southern Spain

**DOI:** 10.1002/fsn3.1211

**Published:** 2019-10-28

**Authors:** Carmen Maria González, Lorena Martínez, Gaspar Ros, Gema Nieto

**Affiliations:** ^1^ Department of Food Technology, Nutrition and Food Science Veterinary Faculty University of Murcia Murcia Spain

**Keywords:** antioxidant capacity, healthy food, Mediterranean diet, natural antioxidants

## Abstract

The objective of this study was to determine the antioxidant capacity of a Mediterranean diet consisting typical dishes from southern Spain. For that, a 5‐day diet was developed with typical dishes of Murcia. The antioxidant capacity of the diet was measured using ORAC_HF_, FRAP, and DPPH methods, and the total phenolic compound content of this diet was estimated by Folin–Ciocalteu. About 50% of the antioxidant capacity and 29% of the phenolic compounds per day came from sample 14 (artichokes with ham), whereas only 1% and a 4%, respectively, proceed from sample 16 (gypsy pot). The total antioxidant capacity of the diet was estimated as 9,506.33 ET/100 g/person/day by the ORAC_HF_ method, and the total phenolic consume was estimated as 1,839.05 mg GAE/person/day. The proposed diet can be considered an excellent strategy for improving the nutritional status of the population because of its high antioxidant activity and phenolic compound content.

## Graphical abstract

Mediterranean diet is rich in antioxidant compounds as vitamin C, vitamin E, carotenoids, phenolic compounds, and trace elements as selenium or zinc. The total antioxidant capacity of the diet was estimated as 9.506,33 ET/100 g/person/day, and the total phenolic consume was estimated as 1.839,05 mg GAE/person/day. Mediterranean diet is an excellent strategy in order to improve the nutritional status on population.

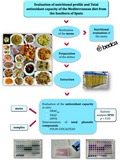

## 1 INTRODUCTION

The term Mediterranean diet (MD) refers to the diet of people who lived in the olive growing areas of the Mediterranean basin between 1950 and 1960 (Trichopoulou et al., 2014). Nevertheless, within the Mediterranean regions there are variations, as in Italy, France, Lebanon, Morocco, Portugal, Spain, Syria, Tunisia, or Turkey (Willett et al., 1995), because it is not simply a diet, but also a lifestyle, with a cultural, social, territorial, and environmental character (Varela Moreiras, 2014). In 2010, UNESCO recognized MD as Intangible Cultural Heritage of Humanity (Rizza, Gara, Antonelli Incalzi, & Pedone, 2016).

The Region of Murcia is situated in southeastern Spain, close to the Mediterranean Sea, it is characterized by its Mediterranean dietary pattern, consisting of a typical and varied gastronomy, whose dishes made from seasonally fresh and minimally processed products, are composed by ingredients rich in natural antioxidants. In the group of fruits and vegetables, pepper and tomato, which are rich in vitamin A, are basic elements of the Murcian cuisine (Márquez, Yépez, Sútil‐Naranjo, & Rincón, 2002) and C (WHO, 2004). Others important fruits are plum, apricot, peach, flat peach, figs, and citrus fruits such as lemon, orange, and tangerine, also rich in vitamin A (Márquez et al., 2002). Legumes are very important in this culture, especially chickpeas, lentils, and broad beans, which are rich in zinc (WHO, 2004). Because of its coastal nature, fish and seafood such as prawns, red mullet, sea bream, mullet, and cod, which are major sources of selenium, are widely consumed (WHO, 2004). The typical meats consumed are rabbit, pork and lamb, as well as Spanish sausages obtained from these meats. Such meat products are rich in vitamin A (Márquez et al., 2002), selenium and zinc (WHO, 2004).

According to the results obtained in recent epidemiological studies, a diet rich in vegetables and fruits is related to a lower risk of chronic diseases (Lampe, 1999). The Mediterranean diet is rich in antioxidant compounds, such as vitamin C, vitamin E, carotenoids, and phenolic compounds, and trace elements such as selenium or zinc (Guo, 2013).

Antioxidants can be classified, according to their origin into endogenous, those which are synthesized in the cell, and naturals, when they are part of the food, such as vitamin C, vitamin E, carotenoids, phenolic compounds, and oligoelements like selenium and zinc (Guo, 2013). Vitamin E is present in vegetable oils (soybean, corn, cotton oil, and safflower) and their derivatives (margarine and mayonnaise), nuts, cereals, legumes, dairy milk, and green leafy vegetable (Sies & Stahl, 1995; Stahl & Sies, 1997). The main dietary sources are fruits and vegetables, especially citrus fruit rich in Vitamin C, ascorbic acid, or ascorbate (WHO, 2004). Carotenoids are fournd in orange or yellow fruits and vegetables, such as mango, melon, red pepper, tomato, or carrot (Márquez et al., 2002). Phenolic compounds can be found in fruits (citrus, strawberries, plums, grapes, olive), vegetables such as eggplant, red cabbage, or radish (Creus, 2004), seeds, flowers, beer, wine, green tea, black tea, and onion (Martínez‐Flórez, González‐Gallego, Culebras, & Tuñón, 2002; Hajlaoui et al., 2019; Martínez, Bastida, Castillo, Ros, & Nieto, 2019; Nieto, Díaz, Bañón, & Garrido, 2010; Nieto, Estrada, Jordán, Garrido, & Bañon, 2011; Nieto & Ros, 2015; Serrano, Ros, & Nieto, 2019; Seraoui, Benkiniouar, Akkal, Ros, & Nieto, 2018). As regards minerals with antioxidant properties, selenium is present in fish, shellfish, meat, eggs, cereals, or dairy milk (WHO, 2004), while the main dietary sources of zinc are meat, cereal, and legumes (WHO, 2004).

Previous studies have evaluated the antioxidant and nutritional value of various typical diets from different parts of the world. In the case of Finnish population, the intake of polyphenols was estimated as 863 ± 415 mg/day, based on individual food consumption records, mainly from berries, fruits, vegetables, grain products, and beverages (Ovaskainen et al., 2008).

Mexico is now the second country in the world for obesity in the adult population and fourth for obesity in children (Dávila‐Torres, González‐Izquierdo, & Barrera‐Cruz, 2015). Hervert‐Hernández, García, Rosado, and Goñi (2011) observed that the consumption of fruits and vegetables by obese women from Mexico was below the recommended dietary intake for fruits and vegetables; however, the diet provided polyphenols and had an antioxidant capacity similar to those of the Spanish diet.

Saura‐Calixto and Goñi (2006) reported than the Spanish Mediterranean diet is characterized by its high antioxidant capacity (6,014 ± 3,549 µmol ET), beverages being the major source, and to a lesser extent, vegetables and fruits, with a very low contribution from cereals.

To our knowledge, there are no studies on the antioxidant capacity of specific diets containing typical dishes from specific regions of the Mediterranean area. Therefore, the overall aim of the present study was to determine the antioxidant capacity and nutritional profile of the typical gastronomy of the Region of Murcia. Moreover, it was evaluated the phenolic compound content, nutritional profile, minerals, antioxidant compounds and the antioxidant capacity of the typical dishes of Mediterranean diet of the southern Spain.

## 2 MATERIAL AND METHODS

### 2.1 Materials and reagents

The extracts used to measure the phenolic compounds and the antioxidant capacity were purchased from Sigma Chemical Co: phenolic reagent Folin–Ciocalteu, gallic acid (3,4,5‐trihydroxybenzoic acid), TPTZ (2,4,6‐Tri(2‐pyridyl)‐s‐triazine, FeCl_3_·6H_2_O, DPPH (2,2‐Diphenyl‐1‐picrylhydrazyl), Trolox (6‐Hydroxy‐2,5,7,8‐tetramethylchroman‐2‐carboxylic acid), AAPH (2,2′‐Azobis (2‐amidino‐propane) dihydrochloride), and Fluorescein (Na salt).

### 2.2 Diet

A diet for 5 days (Table 1) was elaborated with reference to the “Pyramid of Healthy Eating,” based on a variety of dishes of the Murcian cuisine. Each day was divided into five intakes (breakfast, brunch, lunch, afternoon snack and dinner) and provided 2000 kcal per day, with a macronutrients profile of 15%, 35% fat and 50% carbohydrates.

**Table 1:** Five days diet with typical Murcian gastronomy, based on Mediterranean Diet

	Day 1	Day 2	Day 3	Day 4	Day 5
Breakfast	Coffee with milk. Whole wheat toast with tomato. olive oil and salt	Coffee with milk. Whole wheat toast with tomato. olive oil and salt	Coffee with milk. Whole wheat toast with tomato. olive oil and salt	Coffee with milk. Whole wheat toast with tomato. olive oil and salt	Coffee with milk. Whole wheat toast with tomato. olive oil and salt
Brunch	Yogurt with kiwi	Yogurt with kiwi	Yogurt with kiwi	Yogurt with kiwi	Yogurt with kiwi
Lunch	Lettuce salad. Lentil stew. Orange with cinnamon	Murcian´s gazpacho. Rice and vegetables with cod. Fruit salad	Salmorejo. Gilthead bream to salt with grilled vegetables. Pears with red wine	Murcian salad. Gypsy pot. Orange with cinnamon	Hummus. Cod meatball stew. Fruit salad
Afternoon snack	Coffee with milk. Whole wheat toast with tomato. olive oil and salt	Coffee with milk. Whole wheat toast with tomato. olive oil and salt	Coffee with milk. Whole wheat toast with tomato. olive oil and salt	Coffee with milk. Whole wheat toast with tomato. olive oil and salt	Coffee with milk. Whole wheat toast with tomato. olive oil and salt
Dinner	Boiled beans. Yogurt with kiwi.	Pisto murciano. Whole wheat bread. Yogurt with kiwi.	Zarangollo. Whole wheat bread. Yogurt with kiwi.	Artichokes with ham. Whole wheat bread. Kiwi.	Country omelette. Whole wheat bread. Yogurt with kiwi.

The databases BEDCA (Spanish Food Composition Database) were used for the development of the diet.

### 2.3 Samples

Twenty‐two different dishes were prepared using, which ingredients bought in a local supermarket (M1: hummus; M2: kiwi; M3: fruit salad; M4: salmorejo (cold tomato soup with garlic and bread); M5: lettuce salad; M6: yogurt with kiwi; M7: orange with cinnamon; M8: whole meal bread toast with tomato, olive oil and salt; M9: pears with red wine; M10: cod meatball stew; M11: rice and vegetables with cod; M12: gilthead sea bream in salt with grilled vegetables; M13: pisto murciano; M14: artichokes with ham; M15: zarangollo (scrambled egg with courgettes and potatoes); M16: gypsy pot (containing chicpeas, white beans and pears); M17: lentil stew; M18: Murcian gazpacho (peeled tomatoes in onion, olives and tunes); M19: boiled greens beans; M20: Murcian salad; M21: country omelette; M22: coffee with milk). The ingredients of the dishes were as follows:

- M1 (hummus) contained chickpeas, toasted sesame seeds, extra virgin olive oil, lemon juice, garlic, parsley, paprika, cumin, black pepper, and carrot.
- M3 (fruit salad) contained pineapple, strawberry, kiwi, banana, and orange juice.
- M4 (salmorejo) contained egg, ham, garlic, tomato, white bread, wine vinegar, iodized salt, and extra virgin olive oil.
- M5 (lettuce salad) contained lettuce, tomato, carot, natural tuna, olive, onion, wine vinegar, iodized salt, and extra virgin olive oil.
- M6 (yogurt with kiwi) contained kiwi and yogurt.
- M7 (orange with cinnamon) contained cinnamon and orange.
- M8 (whole meal toast with tomato, olive oil, and salt) was composed by whole wheat toast, grated tomato, extra virgin olive oil, and iodized salt.
- M9 (pears with red wine) contained pear, red wine, sugar, and cinnamon.
- M10 (cod meatball stew) contained cod, potato, peas, artichoke, tomato, onion, white bread, egg, garlic, extra virgin olive oil, parsley, and iodized salt.
- M11 (rice and vegetables with cod) contained rice, tomato, garlic, peas, cauliflower, artichokes, red pepper, cod, lemon juice, parsley, extra virgin olive oil, and iodized salt.
- M12 (gilthead sea beam in salt with grilled vegetables) contained by gilthead bream, courgette, tomato, aubergine, red pepper, green asparagus, onion, potato, and extra virgin olive oil.
- M13 (pisto murciano) contained tomato, onion, aubergine, courgette, red pepper, green pepper, egg, extra virgin olive oil, and iodized salt.
- M14 (artichokes with ham) was made from artichokes, ham, onion, lemon juice, extra virgin olive oil, and salt.
- M15 (zarangollo) was formed by courgette, onion, egg, extra virgin olive oil, and iodized salt.
- M16 (gypsy pot) was composed by chickpea, potato, pumpkin, bean, pear, tomato, garlic, onion, carrot, turmeric and extra virgin olive oil.
- M17 (lentil stew) was made from lentil, onion, carrot, garlic, serrano ham, potato, courgette, chorizo, tomato, turmeric, black pepper, extra virgin olive oil and iodized salt.
- M18 (Murcian gazpacho) was formed by tomato, green pepper, onion, cucumber, wine vinegar, garlic, extra virgin olive oil, and iodized salt.
- M19 (boiled beans) was composed by potato, bean, cauliflower, carrot, onion, egg, extra virgin olive oil, and salt.
- M20 (Murcian salad) was made from tomato, onion, natural tuna, black olives, egg, and extra virgin olive oil.
- M21 (country omelette) was formed by green asparagus, peas, garlic, egg, extra virgin olive oil, and iodized salt.
- M22 (coffee with milk) was composed by semi‐skimmed milk and soluble coffee.

The liquefied and minced solid samples were stored in 100 ml jars and frozen at −18°C for a week until analysis.

### 2.4 Food antioxidant extraction

The methodology described by Wu, Duckett, Neel, Fontenot, and Clapham (2008) with some modifications was followed up for sample extraction. Each sample was weighed into two grams in falcon tubes of 50 and 18.5 ml of 25% ethanol were added. The tubes were placed on a magnetic shaker (TENZO M‐500) and shaken for 1 hr at 680 rpm. Then, the samples were centrifuged for 4 min at 3,500 *g* at 4°C, using a centrifuge (Kubota Corporation). The final solution obtained after the elimination of the precipitate was filtered through hydrophilic 0.45 µm nylon filters.

### 2.5 Antioxidant activity assays

#### 2.5.1 Hydrophilic ORACassay

The methodology followed was described by Prior et al. (2003). Filtered samples were diluted 100, 250, 500, 750, 1,000, 1,500, 3,500, or 1,000 times with phosphate buffer. Twenty microliters of the diluted sample or blank (phosphate buffer or Trolox (6.25, 12.5, 25, 50 µl) was added to a 96‐well microplate (Costar). To each well 200 µl of fluorescein solution was added, and the microplate was incubated at 37°C for 15 min. Subsequently, 20 µl AAPH solutions were added to each well. With an excitation wavelength of 485 nm and an emission wavelength of 528 nm, the microplate reader (Multi‐Detection Microplate Reader [Synergy™HT]; Biotek Instruments) was programmed to read the fluorescence at 1 min intervals for 1:30 hr using software GEN 5™.

#### 2.5.2 FRAP (ferric reducing antioxidant power) assay

The method described by Benzie & Strain (1996) was followed. First of all, a pattern line was made from a mother solution of 500 µM Trolox, at 10, 50, 100, 250 and 500 µM. In a 96‐well microplate (Costar), 180 µl of the FRAP working solution was mixed with 20 µl of the sample or Trolox solution and the absorbance was read at the absorption maximum of 593 nm, in a microplate reader (Multi‐Detection Microplate Reader [Synergy™HT]; Biotek Instruments).

#### 2.5.3 DPPH (2,2‐Diphenyl‐1‐picrylhydrazyl) assay

The method described by Brand‐Williams, Cuvelier, and Berset (1995) and Sánchez‐Moreno, Larrauri, & Saura‐Calixto (1998) was used to construct a pattern line from the 500 µM Trolox mother solution at 10, 50, 100, 250, and 500 µM. Then, in a similar 96‐well microplate to that described above, 195 µl of DPPH working solution was mixed with 5 µl of the sample or Trolox solution and the absorbance was read at the absorption maximum of 515 nm after 30 min of mixing, in the microplate reader The whole process was carried out protecting the samples from the light in order to prevent oxidation.

### 2.6 Total phenolic assays

#### 2.6.1 Folin–Ciocalteu index (Phenolic Content Quantification)

The method described by Singleton and Rossi (1965) was used to construct the pattern line with different concentrations of gallic acid 0–20–40–60–80–100. Finally, 85 µl of Na_2_CO_3_ and 100 µl of Folin–Ciocalteu reagent were mixed with 5 µl of the sample or gallic acid solution in the 96‐well microplate. After 1 hr of mixing, the absorbance was read at the absorption maximum of 750 nm in the microplate reader. Throughout the process, the samples were protected from the light to prevent oxidation.

### 2.7 Statistic analysis

Data were analyzed with the statistical package SPSS 21.0 (Statistical Package for the Social Science for Window, IBM). The results for the diet and samples were expressed as mean values ± standard deviation, using ANOVA, Tukey's test, and choosing the results that were statistically significant (*p* < .05).

## 3 RESULTS AND DISCUSSION

### 3.1 Antioxidant capacity of samples

The antioxidant compounds (vitamin A, vitamin E, vitamin C, Zinc, and Selenio) and the antioxidant capacity of the twenty‐two samples were evaluated by ORAC_HF_, FRAP, DPPH, and Folin–Ciocalteu. The results are shown in Table 2.

**Table 2:** Antioxidant capacity (ORAC. FRAP. DPPH), phenolic compounds (FOLIN) and antioxidant compounds (Vit A, Vit E, Zinc, and Selenium) of the samples

Samples	VIT A (µg)	VIT E (mg)	VIT C (mg)	Zn (mg)	Se (µg)	ORAC_HF_ (ET/100g)	FRAP (mg ET/100g)	DPPH (mg ET/100g)	FOLIN (GAE/100g)
1	337.91 ± 5.77^b^	1.95 ± 0.01^ghi^	8.55 ± 0.01^p^	0.73 ± 0.01^j^	1.93 ± 0.07^r^	275.56 ± 0.01^v^	36.20 ± 8.01^gh^	**–**	100.96 ± 5.43^gh^
2	3.1 ± 0.1^r^	1.12 ± 0.06^ijk^	59.1 ± 0.1^g^	0.1 ± 0.5^o^	0.6 ± 0.3^t^	495.89 ± 0.01^n^	74.89 ± 20.32^fg^	57,724.12 ± 5,231.14^fg^	65.84 ± 0.63^hij^
3	22.4 ± 0.4^p^	1.32 ± 0.001^hij^	66.3 ± 0.1^e^	0.24 ± 0.01^n^	1.01 ± 0.01^s^	433.22 ± 0.05^p^	121.85 ± 24.33^ef^	34,908.96 ± 3,480.27^fghi^	116.54 ± 5.20^fg^
4	121.7 ± 0.1^i^	2.05 ± 0.01^fghi^	19.3 ± 0.01^m^	0.75 ± 0.01^j^	7.21 ± 0.01^k^	288.61 ± 0.01^r^	**–**	**–**	50.84 ± 6.44^ij^
5	326.37 ± 2.31^c^	1.89 ± 0.01^ghi^	15.93 ± 1.15^o^	0.43 ± 0.01^lm^	17.34 ± 0.04^f^	281.03 ± 0.01^t^	**–**	5,906.64 ± 5,316.21^i^	36.12 ± 0.83^j^
6	83.25 ± 0.01^l^	1.12 ± 0.01^ijk^	61.75 ± 0.02^f^	0.91 ± 0.04^i^	8.6 ± 0.03^i^	707.44 ± 0.06^i^	25.62 ± 3.1^gh^	21,374.55 ± 5,839.01^ghi^	101.26 ± 10.94^gh^
7	92.52 ± 0.05^k^	0.4 ± 0.01^jk^	100.57 ± 0.01^a^	0.39 ± 0.01^m^	2.3 ± 0.03^p^	913.01 ± 0.08^g^	357.5 ± 44.44^bc^	190,748.1 ± 16,904.48^c^	217.51 ± 1.04^d^
8	36.2 ± 0.01^o^	2.31 ± 0.09^efgh^	7.6 ± 0.01^q^	1.34 ± 0.01^f^	25.01 ± 0.07^d^	477.91 ± 0.06^o^	17.94 ± 12.61^gh^	**–**	100.56 ± 7^gh^
9	3.58 ± 0.09^r^	0.01 ± 0.01^k^	5.85 ± 0.03^r^	0.48 ± 0.01^kl^	2.2 ± 0.01^q^	2034.08 ± 0.01^c^	370.84 ± 28.91^bc^	117,662.25 ± 4,392.04^e^	278.9 ± 7.74^c^
10	179.63 ± 0.07^f^	4.62 ± 1.74^a^	44.06 ± 0.01^i^	2.01 ± 0.01^b^	41.47 ± 0.01^b^	1,446.73 ± 0.01^d^	310.11 ± 17.48^c^	179,147.17 ± 25,025.04^cd^	203.62 ± 9.08^d^
11	62.99 ± 0.01^m^	3.14 ± 0.01^p^	58.86 ± 0.01^g^	1.08 ± 0.01^h^	42.72 ± 0.04^a^	4,455.12 ± 0.07^b^	394.75 ± 52.18^b^	242,952.27 ± 28,660.01^b^	355.43 ± 26.40^b^
12	62.17 ± 0.02^m^	4.3 ± 0.04^ab^	80.61 ± 0.06^c^	1.47 ± 0.04^e^	38.46 ± 0.07^c^	638.97 ± 0.02^k^	121.56 ± 3.71^ef^	59,657.61 ± 3,544.14^fg^	152.37 ± 11.18^ef^
13	219.23 ± 0.58^e^	3.72 ± 0.01^abcd^	88.38 ± 0.03^b^	1.06 ± 0.01^h^	7.01 ± 0.01^l^	1,048.55 ± 0.08^e^	167.65 ± 7.31^de^	148,211.36 ± 13,937.21^de^	121.68 ± 8.18^fg^
14	7.8 ± 0.01^q^	2.37 ± 0.01^efgh^	16.85 ± 0.01^n^	1.28 ± 0.07^fg^	7.2 ± 0.01^k^	6,695.43 ± 0.57^a^	553.3 ± 20.50^a^	389,123.97 ± 8,784.08^a^	607.26 ± 42.61^a^
15	104.4 ± 0.08^j^	2.61 ± 0.01^defg^	33.45 ± 0.01^k^	1.23 ± 0.05^g^	6.55 ± 0.06^m^	844.54 ± 0.03^h^	35.47 ± 4.84^gh^	24,854.83 ± 6,797.52^ghi^	175.15 ± 31.66^de^
16	326.71 ± 0.01^c^	4.25 ± 0.01^abc^	48.23 ± 0.01^h^	2.01 ± 0.01^b^	4.48 ± 0.01^n^	142.46 ± 0.01^w^		13,640.6 ± 11,025.96^hi^	102.65 ± 9.76^gh^
17	290.19 ± 0.01^d^	2.82 ± 0.01^cdef^	30.3 ± 0.04^j^	3.65 ± 0.01^a^	14.54 ± 0.04^g^	538.07 ± 0.07^m^	33.73 ± 16.52^gh^	40,709.43 ± 4,182.77^fghi^	88.2 ± 7.63^ghi^
18	84.7 ± 0.01^l^	1.83 ± 0.01^ghi^	33.45 ± 0.05^k^	0.23 ± 0.02^n^	1.06 ± 0.08^s^	287.09 ± 0.06^s^	21.85 ± 26.54^gh^	6,293.34 ± 7,088.28^i^	53.76 ± 2.13^ij^
19	794.41 ± 0.01^a^	3.2 ± 0.04^bcde^	79.89±0.03^d^	1.77 ± 0.01^c^	9.42 ± 0.06^h^	399.23 ± 0.08^q^	34.60 ± 4.58^gh^	12,867.2 ± 3,480.27^hi^	103.48 ± 3.73^gh^
20	135.67 ± 0.05^h^	2.4 ± 0.01^efgh^	18.82±0.01^m^	0.51 ± 0.01^k^	20.18 ± 0.03^e^	604.50 ± 0.07^l^	12.57 ± 4.46^gh^	38,775.94 ± 4,688.45^fghi^	61.4 ± 6.87^hij^
21	167.3 ± 0.01^g^	4.04 ± 0.05^abc^	27.67 ± 0.01^l^	1.57 ± 0.05^d^	7.3 ± 0.01^j^	948.55 ± 0.04^f^	212.57 ± 38.29^d^	52,310.36 ± 2009.33^fgh^	277.93 ± 13.12^c^
22	51.2 5 ± 0.04^n^	0.2 ± 0.08^jk^	7.51 ± 0.01^q^	1.01 ± 0.01^h^	3.7 ± 0.03^o^	685.56 ± 0.06^j^	364.75 ± 13.08^bc^	72,418.63 ± 39,037.3^f^	365.01 ± 19.07^b^

Note

Results are expressed as mean values ± standard deviation. a–v: Different letters in the same column indicate that there are significant differences between the samples (*p* < .05). 1: Hummus; 2: Kiwi; 3: Fruit salad; 4: Salmorejo; 5: Lettuce salad; 6: Yogurt with kiwi; 7: Orange with cinnamon; 8: Whole wheat toast with tomato. olive oil and salt; 9: Pears with red wine; 10: Cod meatball stew; 11: Rice and vegetables with cod; 12: Gilthead beam to salt with grilled vegetables; 13: Pisto murciano; 14: Atrichokes with ham; 15: Zarangollo; 16: Gypsy pot; 17: Lentil stew; 18: Gazpacho; 19: Boiled beans; 20: Murcian salad; 21: Country omelette; 22: Coffee with milk. Vitamin A (VIT A) = µg; Vitamin E (VIT E) = mg; Vitamin C (VIT C) = mg; Zinc (Zn) = mg; Selenium (Se) = µg; ORAC hydrophilic (ORAC_HF_) = ET/100 g (ET = equivalent of trolox); FRAP = mg ET/100 g; DPPH = mg ET/100 g; FOLIN = mg GAE/100 g (GAE = gallic acid).

As can be observed, the highest ORAC_HF_ value was found for sample number 14 (artichokes with ham), 6.695 43 ± 0.57 µmol TE/100 g, and the lowest for sample 16 (gypsy pot), 142.46 ± 0.01 µmol TE/100 g.

In addition, sample number 14 was considered the most antioxidant, not only for its ORAC_HF_ values, but also FRAP (553.3 ± 20.50 mg ET/100 g), DPPH (389,123.97 ± 87,784.08 mg ET/100 g), and FOLIN (607.26 ± 42.61), which were also the highest recorded. This dish is composed of artichoke, serrano ham, onion, extra virgin olive oil, lemon juice, and iodized salt. Of these, artichoke has an important role because of its powerful hepatoprotective, anticarcinogenic, and hypocholesterolemic (Llorach, Espin, Tomas‐Barberan, & Ferreres, 2002), which are the result of its high phenolic compound content, principally caffeic acid derivatives and flavonoids (Tomás‐Barberán, Ferreres, & Gil, 2000). However, the significance of these compounds is not only due to their antioxidant properties, but also to their vasodilator, anti‐inflammatory, antibacterial, immune system stimulator, antiallergenic, antiviral, estrogenic, or prooxidant enzyme inhibitor effects (García Gabarra, 2006). These findings reflect the results obtained by Ferracane et al. (2008), who reported the antioxidant capacity of cooked artichokes. Another ingredient of the dish was onion, which is the only tuber that contains flavonoids, mainly quercetin 4′‐D‐glucosides, a compound with a high antioxidant power due to its ability to join biological polymers, hormone transporters and DNA, or chelate metal ions (Fe^+2^, Cu^2+^, Zn^2+^; Martínez‐Flórez et al., 2002). Serrano ham is a traditional product in the Mediterranean area, where it is especially value for its sensory characteristics. Because of its well‐balance nutritional profile, it can be included in a balanced diet, since it can generate beneficial effects in cardiovascular disorders (Jiménez‐Colmenero, Ventanas, & Toldrá, 2010). These benefits may be due to the high antioxidant capacity according to the results obtained by Joaquín Martínez, Nieto, and Ros (2014), who determined the antioxidant capacity of meat products was determined using the ORAC_HF_ assay. Olive oil is a fundamental part of the Mediterranean diet and the principal source of fat therein. It is considered one of the compounds responsible for the beneficial effects of the MD due to its antioxidant capacity. Olive oil is rich in phenolic compounds, particularly in hydroxytyrosol and oleuropein (Visioli, Poli, & Gall, 2002). Moreover, this oil is a natural source of the natural antioxidant, vitamin E, which acts to neutralize singlet oxygen (^1^O_2_), capture hydroxyl radical (OH^•^), capture oxygen (O_2_), and neutralize peroxides (Venereo Gutiérrez, 2002). Finally, lemon juice is rich in vitamin C, whose antioxidant action is due to its ability to neutralize ^1^O_2_, capture OH^•^, capture oxygen O_2,_ and regenerate the oxidized form of vitamin E (Venereo Gutiérrez, 2002).

The least antioxidant sample was nº 16, whose ingredients include potatoes. Several studies have shown that potato contain bioactive compounds with antioxidant properties, such as polyphenols, ascorbic acid, carotenoids, tocopherols, and selenium (Nimse & Pal, 2015; Venereo Gutiérrez, 2002). The ingredients also include tomato, which contains flavonoids, phenolic acids, carotenoids, ascorbic acid, and vitamin E, the last one responsible for the beneficial effects associated with this food (George, Kaur, Khurdiya, & Kapoor, 2004); garlic, whose therapeutical effects (hypolipidemic, antiatherosclerotic, hypoglycemic, anticoagulant, antihypertensive, antimicrobial, and hepatoprotective) may be due to the phenolic compounds and flavonoids it contains (Bozin, Mimica‐Dukic, Samojlik, Goran, & Igic, 2008); carrots, which are a good source of antioxidant compounds containing carotenes, vitamin C and phenolic compounds (Alasalvar, Grigor, Zhang, Quantick, & Shahidi, 2001); turmeric, with a high antioxidant activity due a phenolic compound named curcumin (diferuloylmethane; Kaur & Kapoor, 2002); onion and extra virgin olive oil, whose antioxidant compounds have been mentioned above. Despite containing these ingredients rich in antioxidant and phenolic compounds, the lowest antioxidant capacity value of sample nº 16 is due to the way in which it is prepared (pressure‐cooking), which generates significant losses of up to 50% of the antioxidant capacity (Jiménez‐Monreal, García‐Diz, Martínez‐Tomé, Mariscal, & Murcia, 2009).

Samples 2 (kiwi), 3 (fruit salad), 6 (yogurt with kiwi), 7 (orange with cinnamon), 8 (whole wheat toast with tomato, olive oil and salt), 12 (gilthead sea beam in salt with grilled vegetables), 15 (zarangollo), 17 (lentil stew), 19 (boiled beans), and 20 (Murcian salad) showed intermediate values of ORAC_H,_ ranging from 400 to 800 µM ET/100 g. All these dishes contain vegetables and fruits rich in antioxidant compounds, which have not undergone aggressive cooking. Firstly, samples 2, 3, 6, and 7 contain fruits, such as kiwi (rich in vitamin E, folates, carotenoids and, especially, in vitamin C; López‐Sobaler, Aparicio Vizuete, Anta, & María, 2016) or orange (source of vitamin C and polyphenolic compounds; Klimczak, Małecka, Szlachta, & Gliszczyńska‐Świgło, 2007). Finally, samples 8, 12, 15, 17, 19, and 20 all shared one ingredient with a high antioxidant capacity—olive oil. Furthermore, they contain tomato, onion or potato, all with antioxidant properties. Additionally, taking as reference the results obtained by Martínez et al. (2014), the antioxidant effect of meat products and fish is also important in these samples: serrano ham, spicy pork sausage, gilthead sea bream, or tuna. In the above‐mentioned study, the antioxidant capacity of meat products was determined and compared with fish, vegetables, and milk. This study showed the high antioxidant capacity of meat and meat products compared to other animal products and vegetables. Moreover, the study confirmed that, even though heat treatment results in a lowering of antioxidant capacity, especially in the case of fish, the remaining antioxidant capacity remains significant.

In agreement with Saura‐Calixto and Goñi (2006), there is clearly a need to evaluate the antioxidant power by taking into account all the compounds forming part of the diet, since evaluating the antioxidant capacity of individual compounds tends to overestimate their antioxidant effects.

### 3.2 Antioxidant capacity of the diet

The results obtained for the antioxidant capacity and total phenolic compounds for each day of the diet are shown in Table 3. As can be seen, the highest value for the antioxidant capacity and phenolic compounds is attained on day four, 13,184.01 ± 0.01 ET/100 g and 2,069.1 ± 0.01 GAE/100 g, respectively, while the lowest antioxidant capacity and phenolic compound content are attained on day one, 5,873.18 ± 0.01 ET/100 g and 1,582.22 ± 0.01 GAE/100 g.

**Table 3:** Antioxidant capacity (ORAC) and phenolic compounds (FOLIN) of the diet

	DAY 1		DAY 2		DAY 3		DAY 4		DAY 5	
	ORAC	FOLIN	ORAC	FOLIN	ORAC	FOLIN	ORAC	FOLIN	ORAC	FOLIN
BK	1,163.48	486.69	1,163.48	486.69	1,163.48	486.69	1,163.48	486.69	1,163.48	486.69
BR	707.44	84.45	707.44	84.45	707.44	84.45	707.44	84.45	707.44	84.45
LN	1732.11	336.43	5,175.43	502.26	2,961.66	475.45	1659.96	373.23	2,376.23	419.34
AS	1,163.48	486.69	1,163.48	486.69	1,163.48	486.69	1,163.48	486.69	1,163.48	486.69
DN	1,106.67	187.94	3,053.99	257.46	2,849.98	297.46	8,489.65	638.01	2,953.99	418.43
TOTAL	5,873.18 ± 0.01^e^	1582.22 ± 0.01^e^	11,263.8 ± 0.01^b^	1817.57 ± 0.01^d^	8,846.04 ± 0.01^c^	1,830.73 ± 0.01^c^	13,184.01 ± 0.01^a^	2069.10 ± 0.01^a^	8,364.62 ± 0.01^d^	1895.63 ± 0.01^b^
Total mean ORAC	9.506.33									
Total mean FOLIN	1.839.05									

Note

Results are expressed as mean values ± standard deviation. a–e: Different letters in the same column indicate that there are significant differences between the samples (*p* < .05). ORAC hydrophilic (ORAC) = ETg/L (ET = equivalent of trolox); FOLIN = mg GAE/100 g (GAE = gallic acid).

Abbreviations: BK, Breakfast; BR, Brunch; LN, Lunch; AS, Afternoon snack; DN, Dinner.

González, Periago, and Alonso (2017) determined the average intake of phenolic compounds in Spanish people as 1,365,1 mg/day, which is clearly exceeded by the 1,839.05 GAE/100 g in the case of the diet of Murcia. In the same way, Ovaskainen et al. (2008) estimated the average consumption of phenolic compound in a Finnish population as 863 mg/day, underlining the higher intake of the Spanish population (particulary those who follow a diet typical of Murcia). According to Zamora‐Ros et al. (2013), consuming more than 600 mg of phenolic compounds per day increases life expectancy and the quality of life. Martínez et al. (2014) determined the total antioxidant capacity of a balanced diet should be at least 29,006 µmol ET/day. The total mean antioxidant capacity of the diet studied is 9,506.33 TE/100 g. Hervert‐Hernández et al. (2011) concluded that Mexican rural diet provides a low antioxidant capacity, estimated at around 1000–2000 µmol TE per day due to the low daily intake of fruits and vegetables. The differences in antioxidant capacity and phenolic compounds between these countries, then, seem to be due to the different consumption patterns of each country.

### 3.3 Nutritional evaluation of the diet

Tables 4 and 5 show an evaluation of the macronutrients and micronutrients of each day of the diet and the total for the 5 days. Based on the nutritional objectives established by Bartrina and Majem (2011) for macronutrients, the mean overall intake of carbohydrates, fiber, proteins, total fat, monounsaturated fatty acids (MUFA), polyunsaturated fatty acids (PUFA), saturated fatty acids (SFA), and cholesterol comply with the recommendations. First, the RDA of carbohydrates in a healthy Mediterranean diet is supposed to represent between 50% and 55% of the total energy intake, so that the average consumption of 47.25% represents 94.5% of the recommendation. In the case of fiber, it is advisable to consume more than 28 g per day because of its power to reduce cholesterol levels and cardiovascular risk; the mean consumption of fiber in our diet was 37.31 g, representing 100% of the recommendations (RDA). Protein is supposed to represent 15% of the total energy intake, so that the average consumption of the days (16,97%) again represents 100% of the RDA. Lastly, total fat consumption of a balanced diet is supposed to represent between 30% and 35% of the total energy intake; in our case, therefore, because the mean consumption of fat for all the days analyzed (37.25%), contributes 100% of the fat recommendation intake.

**Table 4:** Evaluation of nutritional profile of the diet

	Energy (kcal)	Carbohydrates (g)	Fiber (g)	Proteins (g)	Total fat (g)	MUFA (g)	PUFA (g)	SFA (g)	Choleste rol (g)
Day 1	1986.32 ± 0.015^c^	208.28 ± 0.03^d^	36.21 ± 0.01^c^	72.69 ± 0.014^d^	88.97 ± 0.01^b^	42.29 ± 0.03^b^	8.57 ± 0.01^d^	19.6 ± 0.01^a^	301.59 ± 0.01^d^
Day 2	1996.33 ± 0.015^b^	234.75 ± 0.01^b^	30.11 ± 0.07^e^	69.34 ± 0.05^e^	74.43 ± 0.05^e^	36.00 ± 0.09^e^	7.79 ± 0.01^e^	16.12 ± 0.01^e^	335.74 ± 0.01^c^
Day 3	1963.34 ± 0.07^e^	289.42 ± 0.05^a^	32.74 ± 0.01^d^	91.59 ± 0.05^b^	86.44 ± 0.01^a^	40.18 ± 0.05^c^	10.61 ± 0.01^a^	19.06 ± 0.01^b^	428.65 ± 0.01^b^
Day 4	1973.61 ± 0.02^d^	214.08 ± 0.01^c^	48.53 ± 0.006^a^	88.23 ± 0.01^c^	82.34 ± 0.05^d^	42.51 ± 0.01^a^	9.83 ± 0.01^b^	16.88 ± 0.01^d^	201.45 ± 0.01^e^
Day 5	2004.68 ± 0.04^a^	204.76 ± 0.01^e^	38.98 ± 0.01^b^	99.25 ± 0.05^a^	82.61 ± 0.04^c^	38.72 ± 0.07^d^	9.75 ± 0.01^c^	17.91 ± 0.01^c^	513.01 ± 0.01^a^
Overall diet	1984.84	230.25 (47.25)	37.31	84.21 (16.97)	82.16 (37.25)	39.94 (18.11)	9.30 (4.21)	17.90 (8.11)	356.08
Recommendation*	2000	50–55	>28	15	30–35	20	5	7–8	<300

Note

Results are expressed as mean values ± standard deviation. a–e: Different letters in the same column indicate that there are significant differences between the samples (*p* < .05). Energy = kcal/día; Carbohydrates = g (%); Fiber = g; Proteins = g (%); Total fatl = g (%); MUFA = g (%); PUFA = g (%); SFA = g (%); Cholesterol = mg. Energy = kcal/día; Carbohydrates = %; Fiber = g/day; Proteins = %; Total fat = %; MUFA = %; PUFA = %; SFA = %; Cholesterol = mg/day.

Abbreviations: MUFA, monounsaturated fatty acids; PUFA, polyunsaturated fatty acids; SFA, saturated fatty acids.

* Nutritional objectives for the Spanish population: SENC (2014).

**Table 5:** Evaluation of micronutrients of the diet

	Vitamins			Minerals	
	Vitamin A (µg)	Vitamin E (mg)	Vitamin C (mg)	Selenium (µg)	Zinc (mg)
Day 1	1766.76 ± 0.015^d^	14.03 ± 0.01^e^	373.78 ± 0.01^b^	97.63 ± 0.06^e^	11.55 ± 0.02^a^
Day 2	660.95 ± 0.01^c^	16.13 ± 0.03^b^	397.59 ± 0.08^a^	125.01 ± 0.01^b^	8.75 ± 0.08^e^
Day 3	557.81 ± 0.03^b^	15.08 ± 0.06^d^	289.92 ± 0.06^e^	127.66 ± 0.07^a^	10.07 ± 0.03^c^
Day 4	788.53 ± 0.02^e^	15.55 ± 0.15^c^	333.71 ± 0.01^c^	103.42 ± 0.01^d^	9.92 ± 0.01^d^
Day 5	985.86 ± 0.06^a^	17.06 ± 0.2^a^	297.19 ± 0.01^d^	124.95 ± 0.07^c^	10.76 ± 0.07^b^
mean	951.97	15.57	338.41	115.74	10.20
RDA	800	12	80	55	10

Note

Results are expressed as mean values ± standard deviation. a–e: Different letters in the same column indicate that there are significant differences between the samples (*p* < .05). Vitamin A = µg; Vitamin E = mg; Vitamin C = mg; Zinc = mg; Selenium = µg.

Abbreviation: RDA, Recommended Daily Allowances.

Moreover, this result also satisfies the recommendations for total fat, MUFA, PUFA, SFA, and cholesterol, as determined by García Alonso (2006). Considering that the 30% and 35% of the total energy intake comes from fat, 20% from MUFA, 5% from PUFA, and a 7%–8% from SFA, this diet described contributes 90.55% of the RDA of MUFA, 100% of PUFA, and an 84.2% of SFA.

As regards micronutrient levels, vitamins A, E, and C, and the minerals selenium and zinc meet recommended daily amount established in Spanish regulation (RD1669/2009). Furthermore, both vitamin and mineral levels agree with the maximum tolerable intake level determined by Mathioudakis (2015). The total mean intake of vitamin A suprasses the RDA and so covers 100% of the recommendations. Another point is that the consumption is below UL levels (Tolerable Upper Intake Level). In the case of vitamin E, the average intake was 15 mg, compared to the recommended 12 mg, so that the 100% of the RDA of this vitamin is covered with the diet. Nevertheless, this level is below the UL level. Vitamin C intake was well above the recommendations (338 mg compared with the recommended 80 mg), again contributing 100% of what is needed, although the UL level has not been established for this vitamin. The level of selenium was above that recommended, with a range from 55 to 115 µg, but, at the same time, it was far below the UL; in other words, the diet covered the 100% of the RDA. Finally, the RDA of zinc (10 mg/day) was covered by the diet, and, so once again, this represents the 100% of the RDA.

## 4 CONCLUSIONS

Using different methods (ORAC_HF_, FRAP, DPPH) to evaluate the antioxidant activity in vitro and the Folin–Ciocalteu method to evaluate the phenolic compound content, the results of this study show that the samples corresponding to typical dishes of the Murcia cuisine have a high antioxidant capacity and are rich in phenolic compounds, which contrasts with the results of a previous study evaluating individual ingredients. This could be due to the synergy of the compounds. Thus, the antioxidant capacity and phenolic compounds of the diet of the Region of Murcia suggest that this type of diet could have beneficial effects for health by reducing oxidative damage. In addition, the nutritional properties of the diet—rich in zinc and selenium, with a hight intake of carbohydrates with a low glycaemic index, low in fats, moderate levels of proteins—meet the nutritional objectives of Spanish Society of Community Nutrition (2011).

## CONFLICT OF INTEREST

The authors declare that they do not have any conflict of interest.

## ETHICAL APPROVAL

This study does not involve any human or animal testing.
